# Needs with Regard to Decision Support Systems for Treating Patients with Incurable Non-small Cell Lung Cancer

**DOI:** 10.1007/s13187-019-1471-8

**Published:** 2019-01-26

**Authors:** Dóra Révész, Ellen G. Engelhardt, Johannes J. Tamminga, Franz M. N. H. Schramel, Bregje D. Onwuteaka-Philipsen, Ewoudt M. W. van de Garde, Ewout W. Steyerberg, Henrica C.W. de Vet, Veerle M. H. Coupé

**Affiliations:** 1 Department of Epidemiology and Biostatistics, Amsterdam Public Health Research Institute, VU University Medical Center, De Boelelaan 1089a, PO Box 7057, 1081 HV Amsterdam, The Netherlands; 2 Department of Lung Diseases and Treatment, St. Antonius Hospital, Koekoekslaan 1, 3435 CM Nieuwegein, The Netherlands; 3 Department of Public and Occupational Health, Amsterdam Public Health Research Institute, VU University Medical Center, PO Box 7057, 1081 HV Amsterdam, The Netherlands; 4 Department of Clinical Pharmacy, St. Antonius Hospital, Koekoekslaan 1, 3435 CM Nieuwegein, The Netherlands; 5 Center for Medical Decision Sciences, Department of Public Health, P.O. Box 2040, 3000 CA Rotterdam, The Netherlands

**Keywords:** Decision support systems, Non-small-cell lung cancer, Surveys and questionnaires

## Abstract

Treatment decision-making for patients with incurable non-small cell lung cancer (NSCLC) is complex due to the rapidly increasing number of treatments and discovery of new biomarkers. Decision support systems (DSS) could assist thoracic oncologists (TO) weighing of the pros and cons of treatments in order to arrive at an evidence-based and personalized treatment advice. Our aim is to inventory (1) TO’s needs with regard to DSS in the treatment of incurable (stage IIIB/IV) NSCLC patients, and (2) preferences regarding the development of future tools in this field. We disseminated an online inventory questionnaire among all members of the Section of Oncology within the Society of Physicians in Chest Medicine and Tuberculosis. Telephone interviews were conducted to better contextualize the findings from the questionnaire. In total, 58 TO completed the questionnaire and expressed a need for new DSS. They reported that it is important for tools to include genetic and immune markers, to be sufficiently validated, regularly updated, and time-efficient. Also, future DSS should incorporate multiple treatment options, integrate estimates of toxicity, quality of life and cost-effectiveness of treatments, enhance communication between caregivers and patients, and use IT solutions for a clear interface and continuous updating of tools. With this inventory among Dutch TO, we summarized the need for new DSS to aid treatment decision-making for patients with incurable NSCLC. To meet the expressed needs, substantial additional efforts will be required by DSS developers, above already existing tools.

## Background

Lung cancer, of which roughly 80–85% is non-small cell lung cancer (NSCLC) [1], is one of the leading causes of cancer mortality worldwide [2]. Especially, incurable NSCLC patients (stages IIIB and IV) have a very short life expectancy with 1-year survival probabilities ranging between 22 and 47% [3]. Palliative treatment in this setting aims to preserve or improve quality of life, lengthen life, or decrease disease burden. Palliation can consist of symptom relief (best supportive care), or can target tumor tissue itself with, for instance, systemic chemotherapy (CT), radiotherapy (RT), targeted therapies (e.g., epidermal growth factor receptor tyrosine kinase inhibitors, EGFR-TKI), or immunotherapies.

Individually tailored palliative cancer treatment is essential to ensure that patients receive the treatment that optimally matches their values and preferences, avoiding under- or overtreatment, and optimally utilizing all available healthcare resources. Decision-making in the palliative phase is complex due to the large heterogeneity in this population with regard to histological tumor type, biomarker profile, treatment response, and the marginal expected treatment benefit and the potentially severe toxicity of systemic therapy. Particularly with the rapid developments and breakthroughs in the fields of genomic technologies and molecular therapies for lung cancer, treatment decisions will become more multi-factorial and personalized, and it will become increasingly important to support thoracic oncologists (TO) in staying up-to-date.

Treatment guidelines are quite extensive and general, and do not allow for individual tailoring of treatment. Decision support systems (DSS), as add-ons to existing guidelines, can assist TO in formulating an evidence-based treatment advice by comparing various treatment options for an individual patient. Such tools ideally weigh the pros (e.g., symptom relief, life lengthening) and cons of treatments (e.g., side effects, loss of quality of life), as well as the cost-effectiveness. DSS can consist of (multiple) prediction models, nomograms, and decision trees, which are often based on patient and tumor characteristics. Multiple DSS have been developed for incurable NSCLC patients [4], such as the prognostic score developed by Florescu et al. [5] and the nomograms developed by Hoang et al. [6]. These tools are developed to decide whether certain treatments bring enough gain in overall or progression-free survival to be worthwhile for patients. Research has shown that oncologists tend to overestimate life expectancy of patients [7, 8], and that they could benefit from using statistical tools providing survival estimates [7, 9–11].

Although there are a large number of DSS available to aid palliative treatment decisions for incurable NSCLC patients [4], it remains unknown whether the needs of TO are met by the existing tools. Our aim was to shed light on the unmet needs and preferences of TO for future tools in this rapidly developing field.

## Methods

### Online Inventory Questionnaire: Development and Dissemination

We developed an online inventory questionnaire (using the Surveymonkey platform). The inventory first covered background information of the respondents, the available DSS and clinical parameters that they use in decision-making, and whether they have access to a palliative care team in the hospital. Next, we inventoried what the decisive reasons are for not using a DSS, whether they express a need for future tools, and what they would require in terms of future DSS for palliative care decision-making for patients with incurable NSCLC. The online inventory questionnaire was disseminated among the members (i.e., 157 TO) of the Section of Oncology (SON) within the Society of Physicians in Chest Medicine and Tuberculosis (NVALT). The research protocol was approved by the ethical committee of the VU University Medical Center and all respondents provided written informed consent.

### Expert Interviews

Respondents who indicated in the inventory that they were willing to provide additional information about their DSS needs were contacted. Semi-structured phone interviews were then used to explore TO’s needs and preferences concerning (future) DSS in more detail. The aim of the interviews was to put the findings from the questionnaire in context.

### Data Extraction and Analyses

Descriptive analyses were performed on the inventory data. The interviewer (DR) extracted key points from the telephone interviews, and incorporated them into the data from the inventory questionnaire.

## Results

### Respondent Characteristics and Use of DSS or Clinical Parameters

**Fig. 1:**
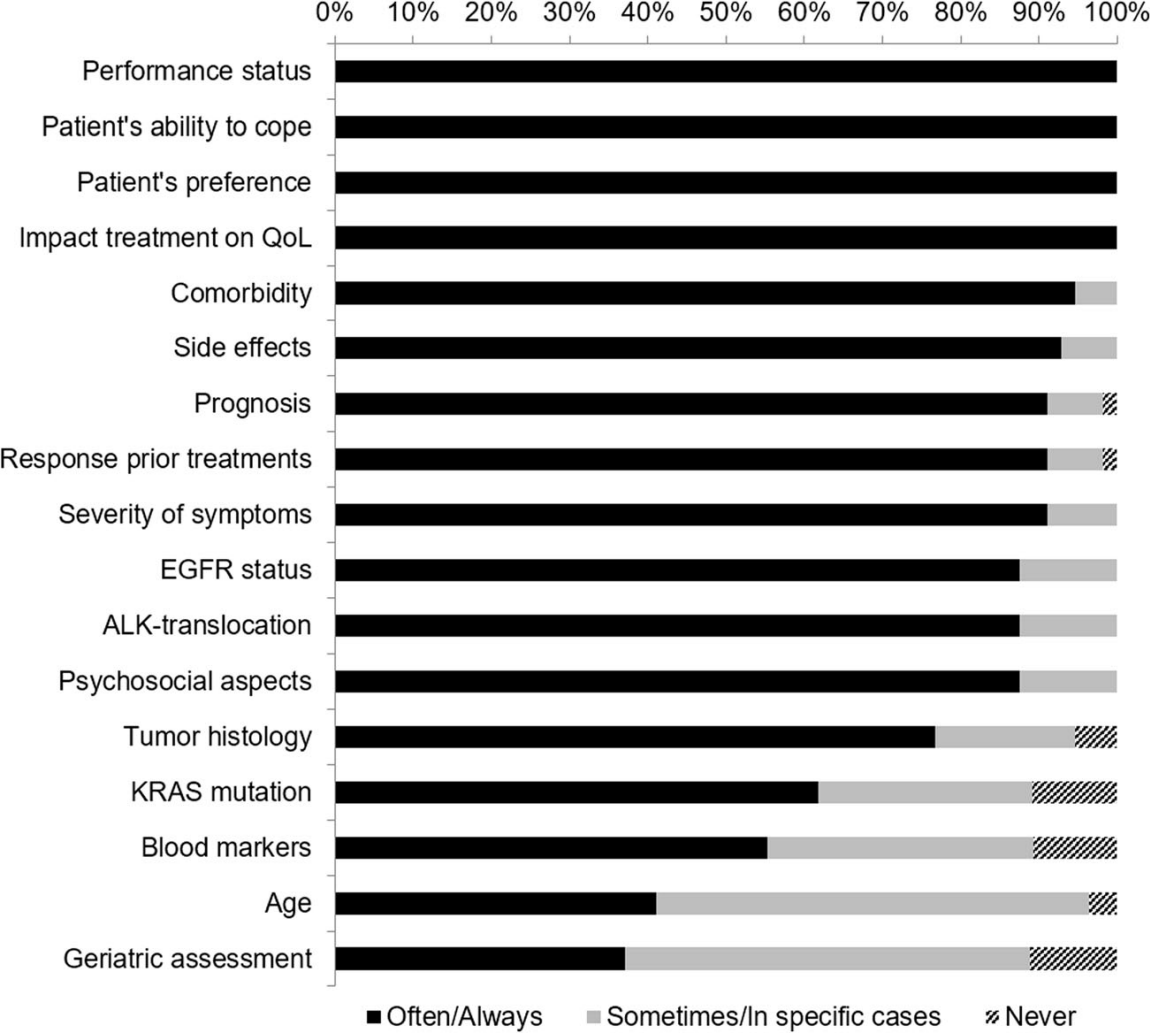
Clinical factors for decision-making in the treatment of incurable NSCLC patients (*N* = 56)

In total, 58 TO filled out the online inventory, of which 37 completed the entire questionnaire (Table 1). The majority of our respondents reported that they treat > 50 incurable NSCLC patients per year, and had > 10 years of experience treating incurable NSCLC patients. Most respondents worked in academic medical centers, and had access to a palliative care team at their hospital. Of the 58 responding TO, 15 were willing to be contacted for in-depth interviews, of which four TO were reached for interviews. In total, 31 TO reported that they make use of an existing tool, although they mostly used clinical guidelines (Table 1). Only few respondents used other DSS, such as the Florescu et al. prognostic score or the recursive partitioning analysis score. Furthermore, they take a wide range of clinical parameters into account during decision-making in their daily practice, such as patients’ performance status, ability to cope, preferences, and the impact on quality of life (Fig. 1).

**Table 1:** General characteristics of thoracic oncologists that completed the online survey (*N* = 58)

Work setting	*N* = 58*
Academic medical centers and teaching hospitals	25 (43%)
General hospital	21 (36%)
University medical center	8 (14%)
Specialized oncological center	3 (5%)
Top clinical care hospitals	1 (2%)
Experience with incurable non-small cell lung cancer patients	*N* = 58
None	0
≤ 2 years	3 (5.2%)
3–5 years	7 (12.1%)
6–10 years	14 (24.1%)
> 10 years	34 (58.6%)
Number of incurable non-small cell lung cancer patients per year	*N* = 58
≤ 5 patients	0
6–10 patients	1 (1.7%)
11–20 patients	1 (1.7%)
21–50 patients	15 (25.9%)
> 50 patients	41 (70.7%)
Access to palliative care team in hospital?	*N* = 58
Yes	57 (98.3%)
Use of existing DSS?	*N* = 33*
Yes	31 (93.9%)
Dutch national NSCLC guidelines	23 (69.7%)
Dutch national palliative care guidelines	16 (48.5%)
Florescu et al. prognostic score	2 (6.1%)
Recursive partitioning analysis score	2 (6.1%)
National Comprehensive Cancer Network guidelines	2 (6.1%)
Hoang et al. nomograms for survival	0
Supportive and Palliative Care Indicators Tool (SPICT)	0
Need of new DSS?	*N* = 51*
Yes	39 (76.5%)
Yes, for shared decision-making	20 (39.2%)
Yes, for treatment decision-making	4 (7.8%)
Yes, for decision-making and shared decision-making	15 (29.4%)

*Numbers can vary due to different amounts of responders, and they do not always add up to 100% as multiple answers were allowed

### Needs and Preferences for (Future) DSS

**Fig. 2:**
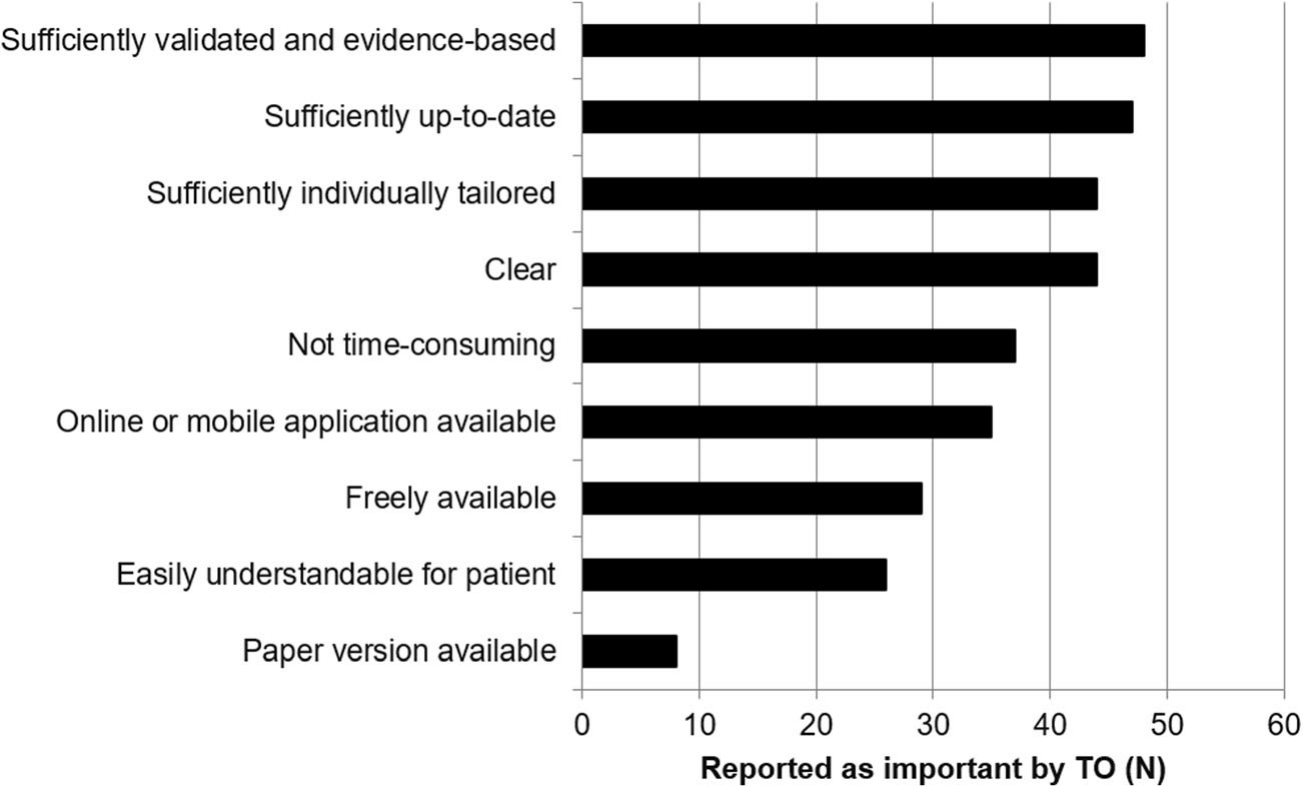
Factors reported to be important for the choice of using certain DSS by TO (*N* = 51)

With the online inventory and the in-depth interviews, we aimed to shed light on the needs and preferences of TO who treat incurable NSCLC patients. Figure 2 summarizes the factors that have been reported to determine whether they use an existing DSS or not. Firstly, DSS should be sufficiently validated and evidence-based, up-to-date (requiring regular updates), and should not be time-consuming. Furthermore, DSS should be easy to use with a clear interface, easy to explain to patients, and freely available, preferably as an online or mobile application.

**Fig. 3:**
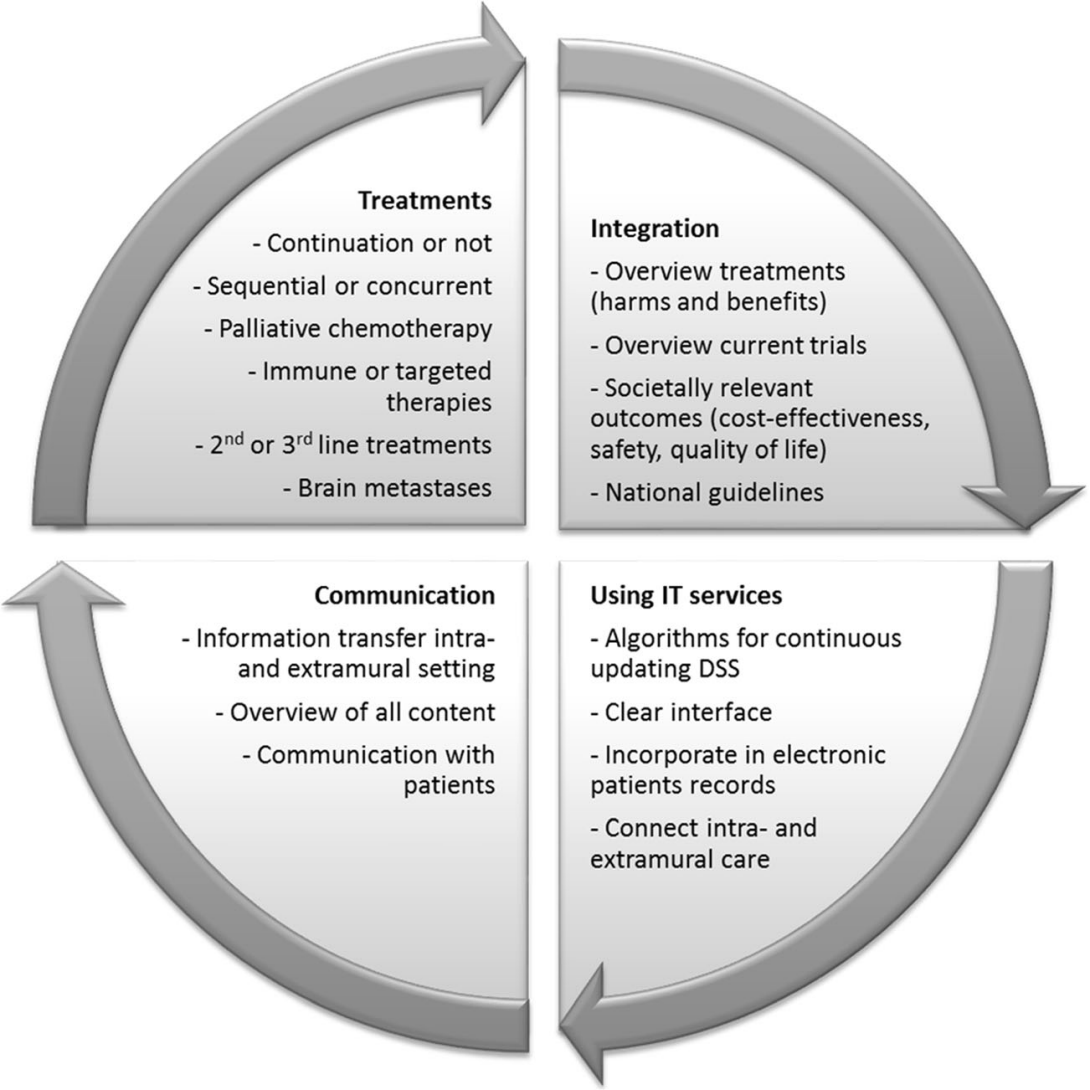
Needs and preferences of TO grouped into the categories Treatments, Integration, Communication, and Information Technology (IT) that can be used for development of future DSS

In addition to the existing tools, 39 TO reported that they would welcome new DSS for their own decision-making and/or for shared decision-making (Table 1). When we asked TO about the specific preferences for future DSS, there was a broad variety of unmet needs reported. These are summarized in Fig. 3, and classified into the aspects “treatments,” “integration,” “communication,” and “using IT solutions.” Firstly, needs were expressed for specific treatment decisions, such as the continuation or discontinuation of specific therapies. Also, future DSS should integrate different treatment options, tailored to the individual patient in a clear overview. These tools should then not only focus on benefits (such as survival), but also the harms of treatment (e.g., toxicity), and on other societally relevant outcome (such as cost-effectiveness). Furthermore, DSS should function as communication enhancers, both between professionals from different disciplines and with patients. Lastly, information technology (IT) can aid by (1) creating clear interfaces, and (2) facilitating information provision to patients about the expected treatment benefits and the side effects of the various treatment options. Also, IT can support future DSS by offering sophisticated algorithms for continuous updating of DSS, and by, for example, incorporating DSS into the electronic patient files. Especially in the telephone interviews, TO mentioned that DSS should not lead to an automated decision-making process, but merely present the advantages and disadvantages of the available treatment options. In other words, oncologists ought to maintain a central role in clinical decision-making.

## Discussion

Online inventory questionnaires were filled out by 58 Dutch TO about their needs and preferences regarding DSS for the treatment of incurable NSCLC patients. The majority of the respondents reported that there is a need for newly developed, up-to-date and validated DSS. Based on the results of the current inventory, recommendations can be made for future DSS development. For optimal use in clinical practice, such tools should integrate all relevant treatment options, weigh the benefits and harms of treatments, facilitate communication between caregivers and with patients, and be kept up-to-date.

To support TO’s decision-making, DSS are a useful and time-efficient complement to clinical guidelines for treatment decision-making. Guidelines could even incorporate DSS that are ready for use in clinical practice. Currently, a large number of DSS have been developed for this population, as shown in our previously published systematic review [4]. Even though the TO in our sample used some of these existing DSS, they reported a need for new tools in their daily practice for multiple goals. Firstly, they missed DSS that show for each individual patient which (combination of) treatments are appropriate, and what the estimated survival gain and toxicities are for each treatment option. Secondly, data on available treatment options, and knowledge from clinical trials, real-life data, and guidelines should be integrated into a DSS. Thirdly, the communication among the different caregivers and with patients could be improved. Lastly, IT solutions were deemed essential, as the challenge will be to design methods for continuous updating of DSS. Moreover, creating clear interfaces for future DSS would make them more suitable to assist in shared decision-making.

Various separate factors, such as performance status, ability to cope, preferences, and the impact on quality of life were reported to be important in clinical decision-making. These are often already incorporated in DSS, but are measured relatively subjectively. Future DSS will need more objective markers, such as genetic and serum markers, in order to reduce unwanted treatment variety and correct use of expensive medication. In the past decades, great progress has been made in the understanding of underlying biological processes of cancer cells. This has led to the discovery of several onco-pathways, which are controlled by oncogenes and tumor suppressor genes. Biological markers are becoming highly influential prognostic and predictive factors for clinical decision-making, as they determine the effectiveness of targeted and immune therapies. In adenocarcinoma and squamous cell carcinoma, various important onco-pathways (e.g., mitogen-activated protein kinase and phosphoinositide 3-kinase pathways) [12, 13], oncogenes (e.g., *KRAS*, *EGFR*, *BRAF*), tumor suppressor genes (e.g., *TP53*, *STK11*, *CDKN2A*), and gene rearrangements (e.g., *ALK*, *ROS*, and *RET*) have been found to be important [14–16]. Furthermore, angiogenesis and the vascular endothelial growth factor affect the processes of primary tumor growth, proliferation, and metastasis [17]. In addition, the immune checkpoints programmed cell death protein-1 and programmed cell death ligand-1 pathways have recently emerged as important targets for immunotherapy [18, 19]. Currently, existing DSS are not up-to-date with regard to such biological markers. Therefore, future tools should incorporate genetic tumor profiles and serum markers to get optimal selection of patients for the myriad of treatment options available. Currently, various national and international guidelines mainly include information about certain genetic mutations of, for example, *EGFR* and *KRAS*, or rearrangements of, for example, *ALK* and *ROS1* [20–23], but there is not a clear decision tool that is tailored to individual patients.

To overcome the reported limitations and to create an adequate tool for future clinical practice, various steps have to be undertaken. It is obvious that oncologist cannot keep up with the current literature to choose the optimal evidence-based treatments, based on a balance between survival gain, side effects, quality of life, patient preferences, and even cost-effectiveness. In addition, the wide array of factors that influence clinical decision-making renders it too complex for the human cognitive capacity. In general, guidelines based on systematic reviews that summarize the evidence of pros and cons of the various treatment options are very useful. Regular updates of the existing guidelines are necessary, but apart from these, DSS can give guidance to decide on the optimal treatment of individual patients. Using the infrastructure of electronic health records for clinical DSS could support personalized medicine practices by providing the tailored information at the right time. Various researchers have discussed the advantages of incorporating DSS in electronic health records in combination with smart algorithms, such that continuous updating and rapid adaptation of DSS are ensured [11, 24–27]. This precision oncology should be a multidisciplinary effort incorporating information from various experts and stakeholders [24, 25].

There are some ethical issues that were raised in conversations with TO, which should be kept in mind as well. A DSS can support the oncologist in giving treatment advice based on the best available evidence. If the DSS is available online and easy to understand, the given information about pros and cons of treatments can be visualized for patients. Nevertheless, this decision-making process should not be automated, even when good DSS become available; oncologists remain essential in the decision-making process. Furthermore, although the oncologist is offering the best treatment options based on available evidence and clinical knowledge, ultimately, the patient decides. However, patients might not feel capable of choosing between the available treatment options.

The strengths of this study were the fact that we had access to the entire Section of Oncology (SON) of the group of Physicians in Chest Medicine and Tuberculosis organization (largest TO organization in the Netherlands), and these TO were distributed over various hospital settings. Considering the fact that the vast majority of our sample used existing DSS, and reported a need for new tools, it is likely that TO with unmet needs have been more inclined to participate than the oncologists who have less knowledge about or need for DSS. Nevertheless, the current group of responders provided valuable insight in TO’s needs and preferences for new DSS.

## Conclusions

In conclusion, TO in the palliative settings reported a need for newly developed DSS when it comes to clinical decision-making in the palliative phase of NSCLC. DSS can be an aid in clinical practice by summarizing and visualizing the current fast-moving area of biomarker-based palliative lung cancer treatments. Future DSS should include novel important prognostic and predictive factors, such as genetic and immune markers, and provide estimates for both survival rates, toxicity, and cost-effectiveness of the available treatments. Future DSS developers should find methods to ensure the continuous updating of DSS and their implementation into daily clinical practice.
